# A Comprehensive Immunocapture-LC-MS/MS Bioanalytical Approach in Support of a Biotherapeutic Ocular PK Study

**DOI:** 10.3390/ph17020193

**Published:** 2024-01-31

**Authors:** Lin-Zhi Chen, David Roos, Elsy Philip, Emily G. Werth, Stephanie Kostuk, Hongbin Yu, Holger Fuchs

**Affiliations:** 1 Boehringer Ingelheim Pharmaceuticals, Inc., Ridgefield, CT 06877, USAelsy.philip@boehringer-ingelheim.com (E.P.); stephanie.kostuk@boehringer-ingelheim.com (S.K.);; 2 Boehringer Ingelheim Pharma GmbH & Co. KG, 88397 Biberach an der Riss, Germany; holger.fuchs@boehringer-ingelheim.com

**Keywords:** anti-drug antibody, anti-peptide antibody, immunocapture-LC/MS, surrogate peptide, albumin, half-life extension

## Abstract

BI-X, a therapeutic protein under development for the treatment of human ocular disease via intravitreal administration, binds to its therapeutic targets and endogenous albumin in the vitreous humor. A monkey ocular pharmacokinetic (PK) study following BI-X administration was conducted to measure drug and albumin levels in plasma, the vitreous humor, the aqueous humor, and retina tissue at various timepoints post-dose. A comprehensive bioanalytical approach was implemented in support of this study. Five immunocapture-LC-MS/MS assays were developed and qualified for quantitating BI-X in different matrices, while ELISA was used for albumin measurement. Immunocapture at the protein or peptide level was evaluated to achieve adequate assay sensitivity. Drug and albumin assays were applied for the analysis of the monkey study samples.

## 1. Introduction

In the last 30 years, biotherapeutics have become an important part of modern medicine. Many biotherapeutics have been developed for a diverse range of targets, treating cancer, heart disease, multiple sclerosis, anemia, and rheumatoid arthritis, among others. Several biologics, such as proteins and antibodies, have been approved to treat various ocular diseases. For example, rituximab (chimeric mAb against the protein CD20) and infliximab (chimeric mAb targeting TNF-α) are marketed for inflammatory eye diseases and uveitis [1,2]. Aflibercept (fusion protein targeting VEGF-A, VEGF-B isoforms, and PLGF) [3,4], brolucizumab (a humanized single-chain antibody fragment against VEGF-A) [5], and faricimab (bispecific mAb targeting VGEF-A and Ang-2) [6] are marketed for age-related macular degeneration (AMD) and diabetic macular edema (DME). Ranibizumab (Fab fragment of bevacizumab) and bevacizumab (humanized full-length mAb) treat AMD, diabetic retinopathy (DR), DME, and retinal vein occlusion (RVO) [7,8,9,10,11,12]. Despite promising results such as improved visual acuity and reduced macular edema and vision loss, the treatment of many ocular diseases remain unmet, creating a need for novel, next-generation medicines. Biologics for eye disease indications have delivery-related limitations, including absorption/permeability across membranes, poor bioavailability, and in vivo stability [13,14]. A short in vivo half-life requires frequent intravitreal administrations, which can be inconvenient, increase cost, and lower patient compliance. The development of the next generation of biologics aims to improve stability, overcome ocular barriers, sustain release, and lower administration frequency. For instance, an albumin–drug complex has been explored to extend a biologic’s half-life [15].

BI-X is a therapeutic protein that is being developed to treat human ocular disease via intravitreal administration. Once administrated to the eye, BI-X binds to its therapeutic targets in addition to endogenous albumin in the vitreous humor, thereby extending the drug’s half-life (t_1/2_). A cynomolgus monkey pharmacokinetic study was conducted with a single intravitreal dose of 0.25 mg BI-X per eye [16]. Vitreous humor, aqueous humor, retina tissue, and plasma samples were collected at various time points post-dose for the determination of drug levels. Given drug distribution and elimination in the eye and in the circulation, drug levels were expected to be significantly different between the different compartments (matrices). In addition, sample availability varied for each matrix. As such, different bioanalytical assays for different matrices are needed. This paper presents a comprehensive immunocapture-LC-MS/MS bioanalytical approach, including different immunopurifications (at the protein level, the peptide level, or a combination of both) and multiple assay developments for the vitreous humor, the aqueous humor, retina tissue, and plasma in support of this monkey ocular study. In addition, albumin was measured in support of PK evaluation. To the best of our knowledge, this is the first report using both protein- and peptide-level immunocapture-LC-MS/MS in support of therapeutic protein drug development.

## 2. Results and Discussion

A cynomolgus monkey pharmacokinetic study was conducted with a single intravitreal dose of 0.25 mg of BI-X per eye [16]. Vitreous humor, aqueous humor, retina tissue, and plasma samples were collected at various time points post-dose for the determination of drug levels. It was expected that the drug levels would be highest in the vitreous humor, with lower concentrations in the aqueous humor and retina and significantly lower levels in plasma according to the general principles of ocular pharmacokinetics. Based on the results from a BI-X rabbit PK study [17], the BI-X level was anticipated to be in the lower ng/mL to pg/mL range in plasma and in the ng/mL to μg/mL range in the other three matrices.

BI-X, a recombinant nanobody protein with a molecular weight of ~41 kDa, is under development for treating ocular diseases via intravitreal administration in humans. It consists of three binding domains, two toward targets and one for human albumin. Once injected into the eye, it binds to the therapeutic targets and endogenous albumin in the vitreous humor. In the presence of albumin, BI-X is expected to be mainly in an albumin-bound form, thereby extending the vitreous half-life. A previous rat ocular pharmacokinetic study showed that BI-X vitreous concentrations at 24 h post-dose were 10-fold higher when dosed together with human serum albumin than when dosed alone [17]. A similar PK study of rabbits led to a three-fold increase in vitreous half-life [17]. 

The endogenous vitreal albumin concentration is an important parameter for translation to humans. Only limited data about albumin concentrations in normal monkeys are available. Vitreal albumin concentrations in Rhesus monkeys are estimated to be approximately 87 µg/mL [18] and thus markedly lower than those reported in healthy donors (average ~300 μg/mL) and diabetic patients (average ~1600 μg/mL) [19,20,21]. Therefore, endogenous vitreal albumin levels were quantified in all monkeys included in the PK study. 

The goal of the present work was to develop and qualify bioanalytical methods in support of the BI-X monkey ocular PK study. Immunocapture-LC-MS/MS was chosen to quantitate the drug, while an ELISA assay was used for albumin measurement. 

A sensitive, sequential protein and peptide immunocapture-LC-MS/MS assay with a lower limit of quantitation (LLOQ) of 50 pg/mL was developed previously in support of another monkey ocular study with BI-X. The same plasma assay was used in the present study. Details of the plasma assay development and validation are described elsewhere [22]. Reported here are the immunocapture-LC-MS/MS assays for quantitating BI-X in the vitreous humor, the aqueous humor, and retina tissue, as well as ELISA for albumin.

**Figure 1:**
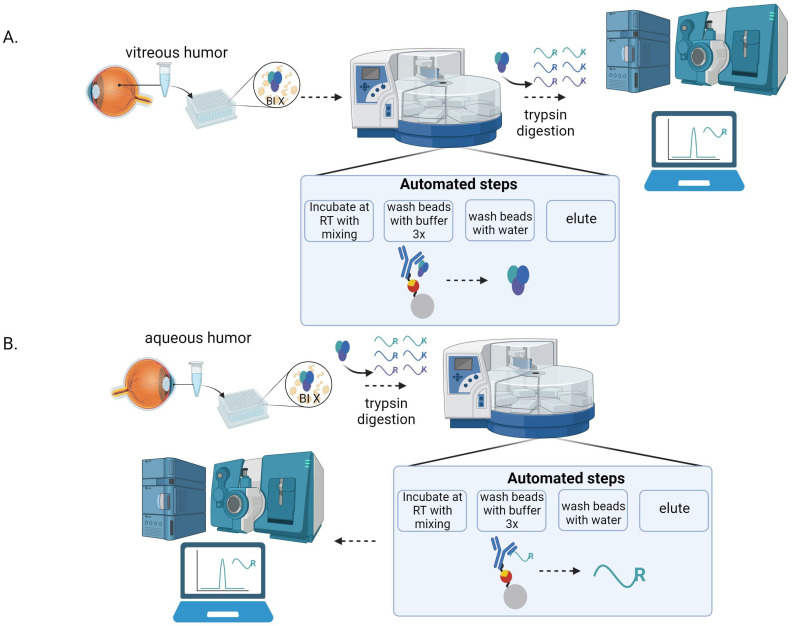
Workflow of immunocapture-LC-MS/MS assays. (**A**) Protein-level immunocapture assay. (**B**) Peptide-level immunocapture assay. Created with BioRender.com.

The immunocapture-LC-MS/MS assays were based on surrogate peptides derived from BI-X since such an approach provides a higher sensitivity than the direct measurement of the whole protein, which typically yields limits of quantitation in the μg/mL range [23,24,25]. The selection of surrogate peptides was based on selectivity, specificity, sensitivity, reproducibility, and robustness, as well as some general selection criteria [26,27]. As reported previously, the tryptic peptides EGVSAIR and YDAVSLEGR, derived from BI-X, met the selection criteria set forth and were thus selected as the surrogate peptides [22]. Depending on the assay, one of the two peptides was used for quantitation, while the other was possibly used for confirmation purposes. Figure 1 shows the workflow of the various immunocapture-LC-MS/MS assays developed in this study.

The peptides were analyzed using a Sciex QTRAP 6500 triple quadrupole mass spectrometer coupled with a micro-flow LC 200 system. Compared with conventional UPLC and nano-flow LC [28], micro-flow LC offers an optimal balance of sensitivity, throughput, and robustness, making it a quite appealing option for the quantitation of proteins [29]. Adequate LC separation was achieved with a reversed-phase Waters BEH C18 column (1 × 50 mm, 1.7 µm). The LC retention time was approximately 2.9 min for EGVSAIR and 3.6 min for YDAVSLEGR. The total run time, including column wash and equilibrium, was 6 min per sample. The MS was operated in positive electrospray ionization [30] and multiple reaction monitoring (MRM) mode and optimized for the detection of the EGVSAIR and YDAVSLEGR peptides. Both peptides were mostly doubly charged in ESI mode and cleaved to singly charged product ions upon collision activation. Product ions with *m*/*z* values higher than their respective parent ions were chosen so that any singly charged interferences would be eliminated from the detection. The most sensitive parent-to-product ion transition was 366.2 → 446.2 for EGVSAIR and 505.1 → 561.3 for YDAVSLEGR. Unit resolution settings on both quadrupoles (Q1 and Q3) were used to minimize the interference peaks while not sacrificing instrument sensitivity.

LC-MS/MS-based quantitation usually requires an internal standard (IS) to compensate for variability during sample preparation and LC-MS/MS analysis. Ideally, a stable isotope-labeled version of the analyte should be used as an IS since it can track the analyte very closely in each step throughout the assay. For the quantitation of small molecules, stable isotope-labeled ISs are routinely used, as they can be easily synthesized. Although stable isotope-labeled ISs should also be used for protein analysis [31], in practice, such ISs are usually not available, especially for proteins that are not mAbs, such as BI-X. However, a rich body of literature has demonstrated that, as long as validation tests demonstrate that the assay is reproducible and robust, there is no preference for the type of internal standard used for protein quantitation [32]. In this study, stable labeled peptides were used as an IS.

### 2.1. Vitreous Humor Assay Qualification

Since BI-X was intravitreally injected into the vitreous humor, drug levels in the vitreous humor are expected to be high initially and then decrease over time. As the study was intended to demonstrate a long vitreal half-life, late timepoints of 10 weeks after dosing were included where low concentrations were expected. To quantitate the drug in such a wide concentration range, two immunocapture-LC-MS/MS vitreous assays were developed: a low-concentration assay and a high-concentration assay with ranges of 1.26–500 ng/mL and 100–50,000 ng/mL, respectively. 

The general workflow of the immunocapture-LC-MS/MS vitreous assays consisted of protein-level immunocapture, immobilization on beads, wash, elution, digestion, and LC-MS/MS detection, as illustrated in Figure 1. A biotinylated mouse mAb against BI-X was spiked to the monkey samples to capture BI-X by forming mAb–drug complexes. After adding streptavidin magnetic beads, the complexes were bound to the beads via biotin–streptavidin interaction. The beads were then separated from the vitreous humor with a magnet. Any residual matrix that remained on the beads was removed by washing them with a buffer. BI-X was then released from the beads upon the addition of 100 µL of 25 mM HCl. The eluent was immediately neutralized to pH 7.5 with 20 µL of Tris-buffer (pH 8.0). 

During the plasma assay development, eight available mouse anti-BI-X mAbs were tested for protein-level immunocapture [22]. The LC-MS/MS peak areas of the surrogate peptides EGVSAIR and YDAVSLEGR were used to evaluate immunocapture efficiency. While all mAbs were found to be able to capture BI-X to different degrees, mAb clone 4E12 provided the highest LC-MS/MS response for both peptides, and EGVSAIR was more sensitive than YDAVSLEGR. Based on the results, mAb 4E12 was selected for immunocapture, and peptide EGVSAIR was used as the surrogate of quantitation for the monkey vitreous humor assay.

The trypsin digestion time was set to 2 h, as no significant gains in the LC-MS/MS signal of the surrogate peptide were attained upon longer digestion. To achieve a maximal sensitivity for the low-concentration assay, a large amount (250 µL) of vitreous humor was used, and 20 μL of the processed sample was injected into the LC-MS/MS system. In contrast, only 10 μL of vitreous sample and a 10 μL injection were used for the high-concentration assay. A stable isotope-labeled IS, EGVSAI[^13^C_6_,^15^N_4_-R], was added to the sample prior to trypsin digestion. After quenching the digestion with formic acid, the samples were injected into the LC-MS/MS system for analysis. To achieve high throughput, the experiments were carried out in 96-well plates, and a KingFisher Flex system was used to automate all the magnetic bead handling steps. EGVSAIR was used for quantitation and YDAVSLEGR was used for confirmation.

**Figure 2:**
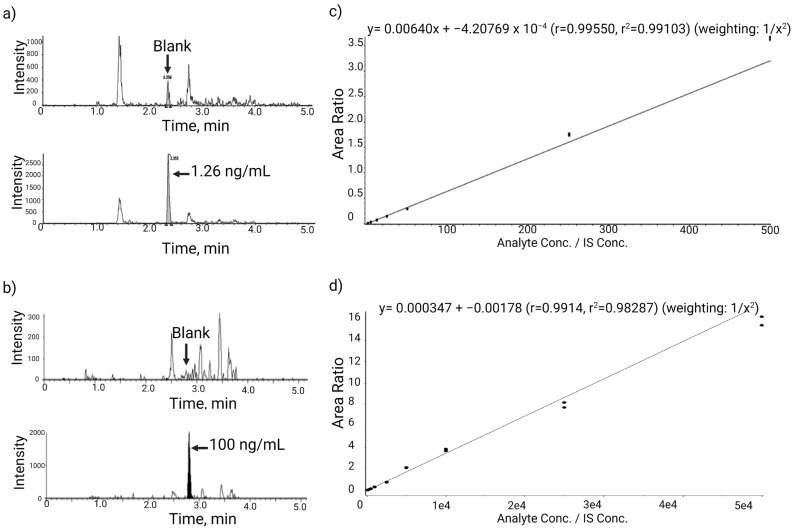
Representative LC-MS/MS chromatograms of blank and LLOQ standards and calibration curves for vitreous low- and high-concentration assays. (**a**) Blank and LLOQ of 1.26 ng/mL calibration standard from the low-concentration assay. Negligible interference peaks were observed in the vitreous blank at retention times of interest for EGVSAIR. There was an interference peak height of 500 cps, far lower than that (4500 cps) of the LLOQ standard. (**b**) Blank and LLOQ of 100 ng/mL calibration standard from the high-concentration vitreous humor assay. (**c**) Calibration curve of vitreous low-concentration curve with a range of 1.26–500 ng/mL. (**d**) Calibration curve of vitreous high-concentration curve with a range of 100–50,000 ng/mL.

The specificity and selectivity of the two vitreous assays were assessed using blank monkey vitreous humor processed following the immunocapture procedures. Figure 2 shows a comparison of LC-MS/MS chromatograms of blank vitreous humor and a 1.26 ng/mL calibration standard (LLOQ) from the low-concentration assay. Negligible interference peaks were observed in the vitreous blank at the retention times of interest for EGVSAIR. An interference peak height of 500 cps is far lower than that (4500 cps) of the LLOQ standard.

Assay linearity, accuracy, and precision were assessed in qualification runs consisting of calibration standards and QC samples. For both low and high-concentration assays, two batches were run on two different days to assess intra-batch (or intra-day) and inter-batch (or inter-day) variability. Calibration standards were injected at the beginning and end of each batch with four replicates of QC sets (a low, a medium, and a high QC per set) in between. The QC levels were 7.49 (low), 30 (medium), and 300 ng/mL (high) for the low-concentration assay and 300, 1000, and 3000 ng/mL for the high-concentration assay. A representative calibration curve obtained during assay qualification for the low-concentration vitreous assay is shown in Figure 2. The assay dynamic linear range was examined by using peak area ratios of the EGVSAIR peptide over the corresponding stable labeled IS (EGVSAI[^13^C_6_,^15^N_4_-R]) from calibration standards and applying a weighted (1/concentration^2^) least-squares linear regression. Table 1 and Table 2 show back-calculated standard concentrations and the linear regression coefficient of determination (*r*^2^). Measured QC concentrations, along with summary statistics of intra- and inter-batch assay precision and accuracy, are provided in Table 3, Table 4 and Table 5.

For non-GLP assay qualification, an individual calibration standard was accepted only if its back-calculated concentration was within ±25% (±30% at LLOQ) nominal concentrations according to company internal guidance. Furthermore, 75% of standards had to meet the acceptance criteria for a calibration curve to be accepted. In total, 14 out of 14 standards for the low-concentration assay and 16 out of 18 standards for the high-concentration assay met the criteria; therefore, all the calibration curves met the acceptance criteria. For the low-concentration assay, mean back-calculated calibration standards were within −10.1% to 10.6% of nominal values (relative error: %RE) at all levels, and coefficients of variation (CVs) were ≤11.5%. For the high-concentration assay, mean back-calculated calibration standards were within −10.7% to 12.7% of nominal values at all levels, and CVs were ≤12.4%. The assay linear range was determined to be 1.26–500 ng/mL, with *r*^2^ ≥ 0.996 for the low-concentration assay and 100–50,000 ng/mL with *r*^2^ ≥ 0.990 for the high-concentration assay.

In each of the two qualification runs, QC samples were assayed to determine drug concentrations. The mean measured concentration of an individual QC, the %RE of the mean QC concentrations compared with the theoretical spiking values, and the CV% were calculated to assess intra-batch and inter-batch assay accuracy (%RE) and precision (CV%). The assay precision must be within ±25% RE, accuracy must be within 25% CV, and at least two out of three QCs and one-half of all QCs at the same level must meet the criteria. For the low-concentration assay, 23 out of 24 QCs at all levels met the acceptance criteria; %RE ranged from −19.6% to 16.2% (intra-batch) and −11.1% to 7.0% (inter-batch), and CV% was ≤12.9% (intra-batch) and ≤14.9% (inter-batch). Likewise, for the high-concentration assay, 24 out of 24 QCs were within ±25% of the spiking concentrations, %RE ranged from 9.8% to 14.8% (intra-batch) and 10.8% to 14.6% (inter-batch), and CV% was ≤7.2% (intra-batch) and ≤6.2% (inter-batch). The QC results demonstrated good intra-batch (or intra-day) and inter-batch (or inter-day) precision and accuracy for both the low- and high-concentration assays.

No significant carryover was observed in blank samples injected immediately after the highest concentration calibration standard.

### 2.2. Aqueous Humor Assay Qualification

Drug levels in the aqueous humor were expected to be lower than in the vitreous body, and a sensitive bioanalytical assay was needed. Usually, higher assay sensitivity can realized by using a larger amount of sample. However, the amount of aqueous humor was limited to approximately 0.13 mL maximum per eye [33]. Many aqueous humor samples from this study had only 50 µL or less. In consideration of sample availability, only a 25 μL aliquot of aqueous humor sample was used in the assay, which presented a challenge for achieving adequate assay sensitivity. We evaluated both protein and peptide immunocapture and found that the latter provided ~2× more sensitivity. Thus, peptide-level immunocapture was selected for the aqueous humor assay.

Like the protein-level immunocapture, the peptide immunocapture approach relied on a quality anti-peptide, Ab. While the YDAVSLEGR peptide was highly immunogenic, the EGVSAIR peptide did not elicit any immune response after primary immunization or three subsequent boost injections. Therefore, only the YDAVSLEGR peptide was suitable for the peptide-level immunocapture approach. Among five available anti-YDAVSLEGR mAbs, clone 9C10 provided the best sensitivity [22] and was selected as the peptide-level capture reagent for the aqueous humor assay. The peptide-level immunocapture was performed after monkey aqueous humor digestion with trypsin, and the assay workflow is shown in Figure 1.

Assay linearity, accuracy, and precision were assessed in two qualification runs, and the results are provided in Table 1, Table 2, Table 3, Table 4 and Table 5. Given the limited availability of blank monkey aqueous humor, rabbit aqueous humor was used as a surrogate matrix to prepare calibration standards and QCs for assay development, qualification, and subsequent study sample analysis. The drug levels in the rabbit aqueous calibration standards ranged from 10 to 50,000 ng/mL, and QC levels were 300 (low), 5000 (medium), and 30,000 ng/mL (high). In addition, small amounts of QC samples at these three levels were prepared in blank monkey aqueous humor. The monkey QCs were assayed along with the rabbit standards and QCs to show parallelism between the two matrices. Matrix could potentially contribute to assay variability in each of the steps, including trypsin digestion, YDAVSLEGR immunocapture, chromatographic separation, and MS detection [34,35]. The establishment of parallelism between rabbit and monkey aqueous humor was essential to ensure that the observed changes in response in the analyte concentration were equivalent for the surrogate and authentic biological matrix across the range of the assay. Furthermore, the IS (YDAVSLEG[^13^C_6_,^15^N_4_-R]) was added prior to the peptide immunocapture step so that it tracked the immunocapture steps in addition to the downstream sample preparation and LC-MS/MS analysis. The stable isotope-labeled IS was able to compensate for sample-to-sample variation in matrix composition, including differences between the two matrices, differences in the peptide immunocapture, and ion suppression/enhancement (which refers to the competition for ionization that occurs between coeluting substances when introduced to the mass spectrometer interface).

**Figure 3:**
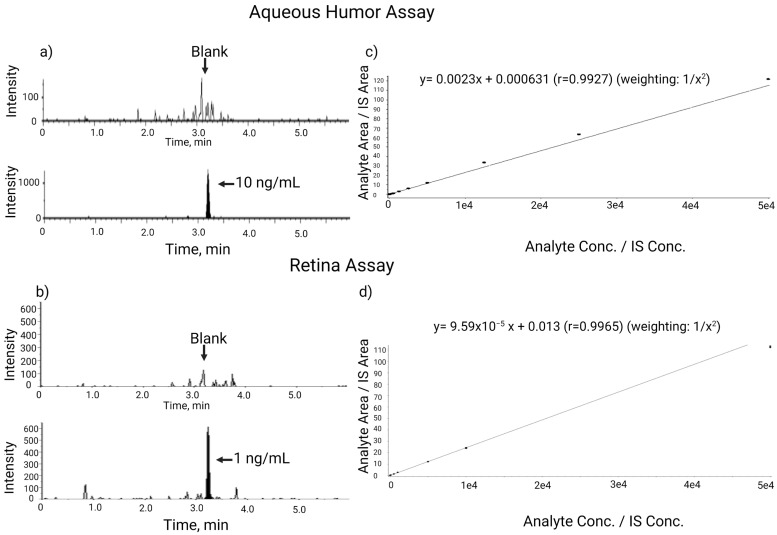
Representative LC-MS/MS chromatograms of blank and LLOQ standards and calibration curve for the aqueous humor and retina homogenate assays. (**a**) Blank and LLOQ of 10 ng/mL calibration standard from the aqueous humor assay. (**b**) Blank and LLOQ of 100 ng/g tissue calibration standard from the retina assay. (**c**) Calibration curve of aqueous humor curve of 10–50,000 ng/mL. (**d**) Calibration curve of retina homogenate curve of 100–5,000,000 ng/g-tissue.

The chromatograms of a monkey blank aqueous humor and an LLOQ standard in rabbit aqueous humor are shown in Figure 3. No interference peaks were observed in the blank matrix at the retention time of the YDAVSLEGR peptide (3.2 min). A typical calibration curve obtained during assay qualification is shown in Figure 3. In the two qualification runs, all 44 individual calibration standards were within ±25% (±30% at LLOQ) of nominal values. The %RE of mean back-calculated calibration standards at all levels ranged from −14.5% to 11.0%, and CV% was from 2.5% to 20.4%, which was within the acceptance criteria. The assay linear range was determined to be 10–50,000 ng/mL with *r*^2^ ≥ 0.992. In total, 22 out of 24 rabbit and 23 out of 24 monkey aqueous humor QCs at all levels met the acceptance criteria (±25%). For the rabbit aqueous humor QCs, %RE ranged from 8.6% to 21.1% (intra-batch) and 9.7% to 18.9% (inter-batch), and CV% was from 2.1% to 8.0% (intra-batch) and 2.2% to 5.7% (inter-batch), demonstrating intra-batch (intra-day) and inter-batch (inter-day) assay precision and accuracy. For the monkey aqueous humor QCs, % RE ranged from 13.8% to 21.4% (intra-day) and 14.7% to 19.0% (inter-day), and CV% was from 2.6% to 7.5% (intra-day) and 4.9% to 5.7% (inter-day). The cyno aqueous humor QC samples demonstrated parallelism between the surrogate rabbit aqueous humor and the authentic monkey aqueous humor.

### 2.3. Retina Tissue Assay Qualification

Like the aqueous humor assay, the peptide-level immunocapture approach was implemented in order to achieve adequate sensitivity in retina tissue. A 50 µL aliquot of the retina homogenate supernatant sample was digested with trypsin, and the resulting YDAVSLEGR peptide and spiked IS (YDAVSLEG[^13^C_6_,^15^N_4_-R]) were captured and enriched using mouse anti-peptide mAb prior to LC-MS/MS analysis. The assay procedures were similar to the aqueous humor assay.

Two qualification runs were conducted to assess assay linearity, accuracy, and precision, and the results are provided in Table 1, Table 2, Table 3, Table 4 and Table 5. The calibration standards and QCs were prepared in blank monkey retina tissue homogenate. The calibration standard concentration ranged from 100 ng/g-tissue to 5,000,000 ng/g-tissue, and QC levels were 300 (low), 25,000 (medium), and 3,750,000 (high) ng/g. Figure 3 shows the chromatograms of a retina blank and an LLOQ standard. In the blank retina, negligible interference peaks were observed. Because of the difficulty in handling tissue and homogenate, ±30% acceptance criteria were used for both calibration standards and QCs. In the two qualification runs, 38 out of 44 individual calibration standards were within ±30% (%RE) of nominal values. The %RE of mean back-calculated calibration standards at all levels ranged from −12.4% to 12.3% (excluding outliers). The CV% ranged from 1.7% to 16.5%. The assay linear range was determined to be 100–5,000,000 ng/g with *r*^2^ ≥ 0.993. A typical calibration curve is shown in Figure 3.

Good intra-batch (intra-day) and inter-batch (inter-day) precision and accuracy were demonstrated in the qualification runs, with 21 out of 24 QCs at all levels within the acceptance criteria (±30%). Intra-batch %RE ranged from −27.3% to 10.0%, and intra-batch CV% was from 3.2% to 16.6%. Inter-batch %RE ranged from −8.0% to 7.1%, and intra-batch CV% was from 6.4% to 23.4%.

### 2.4. Albumin Assay Qualification

Vitreous albumin concentrations from the provided cynomolgus study samples were successfully determined using the internally optimized and qualified commercially obtained ELISA kit method in two accepted analytical runs in which the criteria of the calibration standards and QCs were met. Endogenous QCs were assessed at 6.30 μg/mL (low), 10.12 μg/mL (mid), and 12.60 μg/mL (high). Measurements of nine out of nine low QCs, nine out of nine mid QCs, and nine out of nine high QCs were found within acceptable limits of quantitation (%RE ± 30% of the target concentrations) and (% CV ≤ 30%).

### 2.5. Study Sample Analysis

An ocular PK study was performed to investigate the ocular and systemic pharmacokinetic parameters of BI-X in cynomolgus monkeys after a single intravitreal injection. BI-X was injected bilaterally into the vitreous bodies of the eyes at 0.25 mg/eye. After dosing, plasma, the vitreous humor, the aqueous humor, and the retina were sampled at a series of timepoints using a composite design. Blood (3 mL per timepoint) was collected at 0 (pre-dose), 1, 2, 4, 24, 48, 72, 96, and 168 h and 2, 4, 6, 8, and 10 weeks post-dose. Ocular samples including the vitreous humor, aqueous humor, and retina were collected after animals were sacrificed at 1, 2, 4, 6, 8, and 10 weeks post-dose. A total of 13 monkeys (2 males and 11 females) were used in the study. All the samples were kept at −80 °C prior to analysis. The study details are described elsewhere [16].

The plasma samples were assayed using a sequential immunocapture-LC-MS/MS assay developed previously [22]. The assay range was 0.1–100 ng/mL. Among the 144 plasma samples, 59 with a drug level above the upper limit of quantitation (100 ng/mL) were re-assayed after dilution with blank monkey plasma at a dilution factor of 10. To maintain assay sensitivity and performance at the lower end of the linear curve, dilution QCs and re-assaying samples with high concentrations were used to determine the appropriate solution. This is because the plasma sample volume allowed for the repeat analysis of 59 samples with drug levels above the upper limit of quantitation and the potential distortion in the calibration curve linearity of the lower-concentration measurements if extending the calibration range above the four orders of magnitude used in the 0.1–100 ng/mL assay range. A 10-fold dilution QC was prepared by diluting the high QCs (75 ng/mL) with blank monkey plasma, and they were assayed together with the diluted samples. For all the runs, 49 out of 54 calibration standards and 65 out of 66 QCs, including the dilution QC, met acceptance criteria. All three sample runs met the acceptance criteria. The plasma drug levels in the study samples ranged from BLQ (<100 pg/mL) to 773 ng/mL.

The vitreous humor samples were first assayed using the high-concentration vitreous humor assay. Four samples were above the upper limit of quantitation (50,000 ng/mL) and re-assayed after dilution with blank monkey vitreous humor at a dilution factor of 10. A 10-fold dilution QC was prepared by diluting the high QCs (30,000 ng/mL) with blank monkey vitreous humor, and they were assayed together with the diluted samples. In addition, all samples at weeks 4, 6, 8, and 10 were below the limit of quantitation (100 ng/mL) and were then re-assayed with the low-concentration vitreous humor assay. In total, 18 out of 18 high-concentration calibration standards and 12 out of 12 QCs, including the dilution QC, met the acceptance criteria. In total, 14 out of 18 low-concentration calibration standards and 10 out of 12 QCs met the acceptance criteria. The sample runs met the acceptance criteria. The vitreous humor drug levels in the study samples were in a wide range, from BLQ (<1.26 ng/mL) to 17,400 ng/mL.

The aqueous humor samples were assayed with calibration standards and QCs prepared in rabbit aqueous humor because of a limited supply of monkey aqueous humor. In addition, monkey aqueous humor QCs were assayed in each batch to ensure parallelism between the two matrices. In total, 15 out of 18 calibration standards and 24 out of 24 QCs (including 12 rabbit QCs and 12 monkey QCs) met the acceptance criteria. The sample run the met acceptance criteria. The aqueous humor drug levels in the study samples ranged from BLQ (<10 ng/mL) to 3290 ng/mL, which were all within the calibration curve range (10–50,000 ng/mL).

One run was performed for the retina tissue homogenate sample analysis. The measured drug levels in the homogenate (ng/mL) were converted to the level in retina tissue (ng/g) based on individual tissue weight in the homogenate. No samples were above the limit of quantitation (5,000,000 ng/g tissue). In total, 19 out of 22 calibration standards and 11 out of 12 QCs met the acceptance criteria. The sample run met the acceptance criteria. The drug levels in the retina ranged from BLQ (<100 ng/g-tissue) to 26,446 ng/g-tissue.

The mean albumin concentration of the vitreous humor from all the eyes of the study animals was 34.0 µg/mL, hw mean female albumin concentration was 33.7 µg/mL (*n* = 22), and the mean male albumin concentration was 35.2 µg/mL (*n* = 6). The range of the concentration ratio (right/left eye) was 0.33 to 2.23.

### 2.6. Ocular PK

The ocular pharmacokinetics of BI-X have been discussed and reported previously [16]. Briefly, the ocular pharmacokinetics of BI-X exhibited two phases. The initial phase up to 2–4 weeks after dosing showed BI-X concentrations in the vitreous humor, aqueous humor, and retina declined with half-lives of around 3 days, which is comparable to macromolecules with a similar molecular weight. Thereafter, only vitreal concentrations were measurable, with a terminal half-life of 13.2 days, which is considerably longer than expected based on the BI-X molecular weight or hydrodynamic radius. It is hypothesized that binding BI-X to low levels of intraocular albumin results in this half-life extension. BI-X was detectable in plasma up to 10 weeks post-dosing. The plasma pharmacokinetics of BI-X exhibited a similar biphasic disposition profile to the vitreous body, with a terminal half-life of 11.8 days, thus reflecting input kinetics from the eye. In conclusion, an important half-life extension principle based on vitreal albumin binding could be confirmed in a primate model, and the data obtained could potentially be translated to humans taking into account the differing vitreal albumin concentrations.

## 3. Materials and Methods

### 3.1. Reagent and Materials

BI-X is a proprietary experimental biotherapeutic protein of Boehringer Ingelheim Pharmaceuticals, Inc. (Ridgefield, CT, USA) and was produced in house. A stock solution of BI-X was received at a concentration of 10.28 mg/mL. Surrogate peptides EGVSAIR and YDAVSLEGR and their respective stable isotope-labeled internal standards (ISs), EGVSAI[^13^C_6_,^15^N_4_-R] and YDAVSLEG[^13^C_6_,^15^N_4_-R], were synthesized at Genscript (Piscataway, NJ, USA). Mouse monoclonal anti-BI-X antibody mAb (clone 4E12) was supplied by Precision Antibody, Inc. (Columbia, MD, USA). Mouse anti-peptide YDAVSLEGR mAb (clone 9C10) was produced by Genscript. TPCK trypsin and EZ-Link Sulfo-NHS-LC biotinylation kits were obtained from Thermo Scientific (Rockford, IL, USA). Streptavidin magnetic beads (Magnesphere Paramagnetic Particles) were obtained from Promega (Madison, WI, USA). Blank cynomolgus monkey plasma and cynomolgus vitreous humor, aqueous humor, and retina homogenate samples were purchased from BioIVT (Westbury, NY, USA). Albumin ELISA kits and natural cynomolgus albumin were purchased from Abcam (Cambridge, MA, USA). All other lab chemicals, reagents, and buffer solutions were obtained from Sigma Aldrich (St. Louis, MO, USA), Thermo Scientific, or Invitrogen (Grand Island, NY, USA).

### 3.2. Biotinylation

Biotinylation of the mouse mAbs was performed using an EZ-Link Sulfo-NHS-LC biotinylation kit following the vendor’s instructions. Average biotin incorporation was determined to be 4 biotins per mouse mAb molecule based on HABA assay testing.

### 3.3. Preparation of Standards and Quality Controls

A stock solution of BI-X at a concentration of 10.28 mg/mL was used to prepare all the calibration standards and quality control samples (QCs) in various matrices. The monkey vitreous humor assay calibration standards were prepared in concentrations ranging from 1.26 to 50,000 ng/mL in blank monkey vitreous humor via serial dilution. QC samples were prepared in blank monkey vitreous humor at concentrations of 7.49, 30, 300, 5000, and 30,000 ng/mL.

Aqueous humor assay calibration standards and QCs were prepared using rabbit aqueous humor as a surrogate for monkey aqueous humor because of the availability of blank matrix. Monkey aqueous humor cross-validation QCs were also prepared for analysis against the rabbit standard curve. The assay range was 10 to 50,000 ng/mL with QCs prepared at 30; 5000; and 30,000 ng/mL.

Monkey retina homogenate prepared in chilled RIPA buffer (0.05 g of tissue per 5 mL of buffer) was used to prepare calibration standards and QCs for the retina assay. The calibration standards were prepared at a concentration range of 100 to 5,000,000 ng/g-tissue (equivalent to 1 to 50,000 ng/mL homogenate) from the stock solution via serial dilution. QC samples were prepared at 300, 25,000, and 3,750,000 ng/g tissue in retina homogenate.

The standard albumin calibrator provided with the ELISA kit was replaced with a natural cynomolgus albumin purified from plasma. The lyophilizate was reconstituted to a 10 mg/mL stock. Eight calibration standard points (6.25 ng/mL to 800 ng/mL) were prepared in 1% casein in phosphate-buffered saline (PBS) from the stock using serial dilution. The albumin concentration at 6.25 ng/mL was set as the lower limit of quantitation (LLOQ). Endogenous QCs (QCs with endogenous levels of albumin) were assessed at 6.3 ng/mL (low), 10.12 ng/mL (mid), and 12.6 ng/mL (high). The low and high QCs were obtained from individual lots of cynomolgus vitreous; the mid-QC was obtained from a pooled lot.

### 3.4. BI-X Vitreous Humor High-Concentration Assay

A 10 µL aliquot of monkey vitreous humor sample, 50 µL of 0.1 mg/mL biotinylated mouse mAb against BI-X, 450 µL of Tris-buffered saline with 0.1% Tween-20 (TBS-T), and a 40 µL aliquot of freshly prepared 5 mg/mL magnetic bead solution were added to a 96-deepwell polypropylene plate. The plate was incubated at room temperature for 1.5 h. The beads were separated using a magnet, washed three times with 300 µL of TBS-T and once with 300 µL of water, and then eluted with 100 µL of 25 mM HCl on a Kingfisher Flex magnetic bead handler. The eluent was immediately neutralized with 20 µL of 1 M Tris-HCl (pH 8.0). A 5 µL aliquot of 100 mM TCEP in 100 mM ammonium bicarbonate was added to each sample, and the plate was incubated for 1 h at 60 °C. Next, a 5 µL aliquot of working internal standard solution containing 100 ng/mL of EGVSAI[^13^C_6_,^15^N_4_-R] in water and 5 µL of 200 mM iodoacetamide in 100 mM ammonium bicarbonate were added to each sample. The plate was incubated for 30 min at room temperature in the dark. In total, 5 µL of 100 mM CaCl_2_ and 5 µL of 6 µg/µL trypsin in 50 mM acetic acid were then added to each sample. The plate was incubated for 2 h at 37 °C with gentle shaking. The digestion was quenched with 1 mL of 0.1% formic acid, and the plate was centrifuged at 3800 rpm for 10 min before injection into the LC-MS/MS for analysis.

### 3.5. BI-X Vitreous Humor Low-Concentration Assay

A 250 µL aliquot of monkey vitreous humor sample, 75 µL of 0.1 mg/mL biotinylated mouse mAb against BI-X, 450 µL of Tris-buffered saline with 0.1% Tween-20 (TBS-T), and a 40 µL aliquot of freshly prepared 5 mg/mL magnetic bead solution were added to a 96-deepwell polypropylene plate. The plate was incubated at room temperature for 2 h. The rest of the procedures were the same as the high-concentration vitreous assay.

### 3.6. BI-X Aqueous Humor Assay

A 25 µL aliquot of monkey aqueous humor sample and 25 µL of 8 M urea were added to a 96-deepwell polypropylene plate and mixed at room temperature for 15 min. A 25 µL aliquot of 10 mM TCEP in 100 mM ammonium bicarbonate was added to each sample, and the plate was incubated for 1 h at 60 °C. Next, 25 µL of 20 mM iodoacetamide in 100 mM ammonium bicarbonate was added to each sample, and the plate was incubated for 30 min at room temperature in the dark. A 175 µL aliquot of a solution containing 5 mM CaCl_2_ and 350 µg/mL of trypsin in 100 mM ammonium bicarbonate was then added to each sample, and the plate was incubated with gentle shaking for 2 h at 37 °C. Immunocapture at the peptide level was then started by adding 400 µL of TBS-T, 5 µL of working internal standard solution containing 100 ng/mL of YDAVSLEG[^13^C_6_,^15^N_4_-R] in water, 50 µL of anti-peptide YDAVSLEGR antibody, and 20 µL of freshly prepared 5 mg/mL magnetic bead solution to each sample. The plate was incubated at room temperature for 1.5 h. The beads were separated using a magnet, washed three times with 300 µL of TBS-T and once with 300 µL of water, and then eluted with 100 µL of 25 mM HCl on a Kingfisher Flex magnetic bead handler. The eluent was centrifuged at 3800 rpm for 10 min before injection into the LC-MS/MS for analysis.

As the calibration standards were prepared in rabbit aqueous humor, parallelism between rabbit and monkey aqueous humor was established using the QCs prepared at 30; 5000; and 30,000 ng/mL in monkey aqueous humor and rabbit aqueous humor.

### 3.7. BI-X Retina Tissue Assay

Prior to sample analysis, the retina homogenate samples were gently vortexed and centrifuged for 10 min at 4000 rpm. Sample preparation was performed using a procedure similar to the peptide-level immunocapture described for the aqueous humor assay above. A 50 µL aliquot of the retina homogenate supernatant sample and 50 µL of 8 M urea were added to a 96-deepwell polypropylene plate and mixed at room temperature for 15 min. A 50 µL aliquot of 10 mM TCEP in 100 mM ammonium bicarbonate was added to each sample, and the plate was incubated for 1 h at 60 °C. Next, 50 µL of 20 mM iodoacetamide in 100 mM ammonium bicarbonate was added to each sample, and the plate was incubated for 30 min at room temperature in the dark. A 350 µL aliquot of a solution containing 5 mM CaCl_2_ and 350 µg/mL of trypsin in 100 mM ammonium bicarbonate was then added to each sample, and the plate was incubated with gentle shaking for 2 h at 37 °C. Immunocapture at the peptide level was then started by adding 400 µL of TBS-T, 5 µL of working internal standard solution 100 ng/mL of YDAVSLEG[^13^C_6_,^15^N_4_-R] in water, 50 µL of anti-peptide YDAVSLEGR antibody, and 20 µL of freshly prepared 5 mg/mL magnetic bead solution to each sample. The plate was incubated at room temperature for 1.5 h. The beads were separated using a magnet, washed three times with 300 µL of TBS-T and once with 300 µL of water, and then eluted with 100 µL of 25 mM HCl on a Kingfisher Flex magnetic bead handler. The eluent was centrifuged at 3800 rpm for 10 min before injection into the LC-MS/MS for analysis.

### 3.8. BI-X Plasma Assay

A plasma assay developed for another monkey study with BI-X was used [22]. Briefly, the plasma assay employed an immunoaffinity process consisting of protein-level immunocapture, trypsin digestion, and peptide-level immunocapture in a sequential manner. The protein-level immunocapture procedure was similar to the low-concentration vitreous humor assay described above, except for using 500 μL aliquot of monkey plasma. Following overnight plasma digestion, 325 μL of TBS-T, 5 μL of working internal standard solution of YDAVSLEG[^13^C_6_,^15^N_4_-R] at 1000 ng/mL in water, and 30 μL of 0.1 mg/mL biotinylated mouse anti-peptide antibody were added to each sample. The plate was then incubated at room temperature for 2 h. A 10 μL aliquot of freshly prepared 5 mg/mL magnetic beads was added to each sample, and the plate was gently mixed for 1 h at room temperature. After separating and washing the beads, the surrogate peptide was eluted from the beads and injected into the LC-MS/MS for analysis.

### 3.9. LC/MS

The same LC-MS/MS method was used for the BI-X plasma, high-concentration vitreous humor, aqueous humor, and retina tissue immunocapture-LC-MS/MS assays for the determination of drug levels. An Eksigent Ekspert MicroLC 200 coupled with an AB Sciex 6500 triple quadrupole mass spectrometer (AB Sciex, Framingham, MA, USA) was used. Chromatographic separation was performed using an Acquity Peptide BEH C18 column (1 mm × 50 mm, 1.7 µm, 300 Å) operated at 60 °C. Mobile phases consisted of (A) 0.1% formic acid and (B) 0.1% formic acid in acetonitrile running at a flow rate of 50 µL/min. Sample injection volume was 10–20 μL. The LC gradient was held at 5% B for 1 min and then raised to 30% B over 2.75 min. The column was then washed with 95% B for 1.25 min and re-equilibrated for 1 min before the next injection. The low-concentration vitreous humor LC-MS/MS assay was developed on regular UPLC because of instrument availability at the time when the study was conducted. A Waters ACQUITY UPLC system with an AB Sciex 6500 triple quadrupole mass spectrometer was used. Chromatographic separation was performed using an Acquity Peptide BEH C18 column operated at 60 °C. Identical mobile phase composition and LC gradient, as described above, were used. For the regular-flow UPLC, chromatographic separation was run at a flow rate of 0.3 mL/min until column washing with 95% B, where the flow was increased to 0.6 mL/min. Sample injection volume was 20 μL.

The mass spectrometer was operated in positive electrospray ionization mode. Key instrument parameters were +5500 V electrospray voltage, 50 nebulizer gas units, 30 axillary gas units, 375 °C ion source temperature, 8 collision gas units, and unit resolution on both Q1 and Q3. Multiple-reaction monitoring (MRM) with parent-to-product ion transitions of 366.2 → 446.2 for EGVSAIR, 371.2 → 456.2 for EGVSAI[13C6,15N4-R], 505.1 → 561.3 for YDAVSLEGR, and 510.1 → 571.3 for YDAVSLEG[13C6,15N4-R] were used.

### 3.10. BI-X Assay Qualifications

The BI-X vitreous humor, aqueous humor, retina tissue, and plasma immunocapture-LC-MS/MS assay qualifications involved testing several key parameters, including accuracy, precision, and specificity. This was intended to establish confidence in assay performance for potential further assay validation according to Good Laboratory Practice (GLP). The calibration curves consisted of at least 7 levels, including a blank (containing no drug) and a double blank (no drug, no internal standard), and were analyzed in duplicate. QC samples fortified with BI-X in matrix were tested using 4 replicates at each level in 2 separate batches run on different days. EGVSAIR was used as the surrogate peptide for quantification of BI-X with the protein-level immunocapture procedure (vitreous humor), while YDAVSLEGR was used for quantitation with the peptide-level or sequential immunocapture procedure (aqueous humor, retina homogenate, and plasma). The plasma assay qualification was conducted for a separate study and was reported previously [22].

### 3.11. Albumin Vitreous Humor Assay

Samples were analyzed for albumin concentration using an internally optimized commercially obtained ELISA kit that uses a chromogenic substrate to generate an endpoint readout. The kit provided a pre-coated/pre-blocked plate and a detection antibody reagent. The remaining components of the kit were replaced to improve assay performance. The standard calibrator provided with the kit was replaced with natural cynomolgus albumin purified from plasma. The blocking and detection buffers were changed to improve the selectivity and specificity of the assay.

The kit’s microtiter plates were incubated with 100 µL of cynomolgus albumin reference standard, endogenous quality controls, and samples at a minimal required dilution (MRD) of 1:200 in assay buffer (1% Casein in PBS) for 60 ± 2 min in an incubator set to 22 ± 2 °C. Sample incubation was followed by a 4 × 300 mL wash step with 0.05% Tween 20 in 1X PBS. A 100 µL aliquot of anti-albumin HRP-conjugated detection antibody provided with the kit and diluted to 1 to 100 in assay buffer was then added and incubated for 30 ± 2 min in the dark in an incubator set to 22 ± 2 °C. The wash step was repeated. Detection was performed in the dark with a 10 min bench top incubation of 100 µL of tetramethylbenzidine (TMB) substrate followed by the addition of 100 µL of stop solution. The reaction was allowed to equilibrate and read colorimetrically at 450 nm on a plate reader.

The ELISA assay was qualified to detect albumin in cynomolgus vitreous over an in-well concentration range of 6.25 to 800 ng/mL, with 6.25 ng/mL set as the lower limit of quantitation. Parallelism was established between 1:100 and 1:400 MRD. Assessment of assay parallelism was performed on vitreous from ten individuals, with 8 out of 10 individuals falling within the acceptable limits of quantitation. Dilution linearity was established up to 108 µg/mL.

### 3.12. Ocular PK Study

The in-life portion of the ocular PK study was performed at Boehringer Ingelheim Pharma GmbH & Co. KG, Biberach, Germany, in accordance with the German Animal Welfare Act, and the study was approved by the responsible authority (Regierungspräsidium Tübingen, FRG). The biospecimens, including tissue from this study, were allowed for bioanalysis. In total, 2 male and 11 female cynomolgus monkeys (Macaca fascicularis) were given intravitreal injections of 0.25 mg/eye BI-X (0.5 mg per animal). At the terminal sampling timepoint, the animals were euthanized with an overdose of pentobarbital. Aqueous humor was collected via paracenthesis using a 1 mL insulin syringe and an appropriate cannula. The eye was explanted, and the remaining ocular muscle, retrobulbar fat, and excess conjunctiva were removed. The eyeball was weighed, and the vitreous body was collected via aspiration with a 2.5 or 5 mL syringe without a needle after a circular incision of the sclera at the pars plana. Subsequently, the anterior part of the eye, including the cornea, anterior chamber, iris, ciliary body, and lens, was flipped over, and the remaining vitreous body was collected via aspiration through the luer cone of a syringe without a cannula. Finally, the retina was peeled off, collected, homogenized, and stored at −80 °C. For plasma pharmacokinetics, serial blood samples were taken in EDTA anticoagulant pre-dose and at 1 h, 2 h, 4 h, 24 h, 48 h, 72 h, 96 h, and 168 h and 2, 4, 6, 8, and 10 weeks after dosing or until the last in-life timepoint of the animals. Plasma was prepared and stored at −80 °C in cryovials until bioanalytical measurement. Details of the in-life study were reported elsewhere [16].

## 4. Conclusions

In this cynomolgus monkey ocular PK study with BI-X, plasma, the vitreous humor, the aqueous humor, and the retina were sampled at a series of timepoints for measurements of drug and albumin levels. With a collection of such diverse samples and anticipated wide ranges of drug levels, a comprehensive bioanalytical approach was required. Multiple bioanalytical assays were needed to assay the drug in different matrices. Several immunocapture-LC-MS/MS assays were successfully developed and applied to quantitate BI-X in the vitreous humor, the aqueous humor, retina tissue, and plasma, while an ELISA was developed for the measurement of albumin (Table 6). Precision and accuracy were assessed for each assay, and all met the acceptance criteria. Higher sensitivity was achieved using peptide immunocapture than protein immunocapture, and it was employed for the aqueous humor and retina tissue assays. Sequential immunocapture, which consisted of protein immunocapture followed by digestion and peptide immunocapture, was able to achieve an even higher sensitivity (100 pg/mL) and was used for the plasma assay. Given the wide range of drug concentrations in the vitreous humor, two assays were developed, a low-concentration assay (1.26–500 ng/mL) and a high-concentration assay (100–50,000 ng/mL).

Although different immunocapture approaches were implemented, the same MS analysis method, with either micro-LC or conventional LC for one matrix, was used across all the drug assays. Two tryptic peptides derived from BI-X were monitored, one for quantitation and the other for confirmation. This exemplified the multiplexing capability of LC-MS/MS; i.e., the same method could be applied to different matrices/species, and multiple analytes could be monitored in the same assay.

## Figures and Tables

**Table 1:** Qualification run calibration results of vitreous humor immunocapture-LC-MS/MS assays.

Calibration Standards (ng/mL) of Vitreous Humor Low-Concentration Assay										
	*r* ^2^	1.26	5	12.6	25.1	50.3	251		500	
Batch 1	0.996	1.36	5.34	12.2	23.0	45.1	265		550	
		1.15	5.17	11.9	23.6	45.3	268		556	
Batch 2	0.997	1.24	5.29	12.4	27.8	45.9	254		449	
		1.28	4.60	12.5	28.2	52.3	259		462	
	Mean	1.26	5.26	12.0	23.3	45.2	267		553	
	SD	0.15	0.12	0.21	0.42	0.08	2.73		4.34	
	%CV	11.5	2.2	1.8	1.8	0.2	1.0		0.8	
	%RE	−0.3	5.2	−4.4	−7.1	−10.1	6.2		10.6	
**Calibration Standards (ng/mL) of Vitreous Humor High-Concentration Assay**										
	*r* ^2^	100	250	500	1000	2500	5000	10,000	25,000	50,000
Batch 1	0.990	98.4	219	376 *	845	2000	4960	9380	26,800	49,200
		106	222	385 *	808	2010	4640	9340	27,000	48,000
Batch 2	0.996	101	242	436	953	2440	5290	11,700	30,700	42,900
		107	226	488	972	2480	5400	11,500	28,200	42,800
	Mean	103	227	462	895	2233	5073	10,480	28,175	45,725
	SD	4.09	10.2	36.8	80.3	263	344	1296	1793	3356
	%CV	4.0	4.5	8.0	9.0	11.8	6.8	12.4	6.4	7.3
	%RE	3.1	−9.1	−7.6	−10.6	−10.7	1.5	4.8	12.7	−8.6

* Out of acceptance criteria and not included in statistics.

**Table 2:** Qualification run calibration results of aqueous humor and retina tissue immunocapture-LC-MS/MS assays.

Calibration Standards (ng/mL) of Aqueous Humor Assay												
	*r* ^2^	10	50	125	250	500	1250	2500	5000	12,500	25,000	50,000
Batch 1	0.992	10.1	40.2	113	228	413	1300	2680	5270	14,600	27,500	53,000
		10.9	41.0	104	232	413	1350	2740	5020	14,300	28,800	53,000
Batch 2	0.994	9.46	45.9	106	219	602	1190	2330	4890	12,500	27,600	58,000
		11.2	43.9	114	224	576	1180	2360	4810	13,000	27,100	57,700
	Mean	10	43	109	226	501	1255	2528	4998	13,600	27,750	55,425
	SD	0.79	2.63	4.99	5.56	102	83.5	213	201	1010	733	2803
	%CV	7.6	6.2	4.6	2.5	20.4	6.7	8.4	4.0	7.4	2.6	5.1
	%RE	4.1	−14.5	−12.6	−9.7	0.2	0.4	1.1	−0.1	8.8	11.0	10.9
**Calibration Standards (ng/g Tissue) of Retina Tissue Assay**												
	*r* ^2^	100	250	500	1000	500	1000	5000	10,000	50,000	1,000,000	5,000,000
Batch 1	0.997	99.6	228	664 *	1410 *	5390	9560	43,500	94,600	503,000	1,000,000	4,790,000
		217 *	320 *	571	1080	5370	10,900	49,200	97,600	502,000	*no peak*	4,650,000
Batch 2	0.993	115	203	411	967	5340	12,700	60,200	111,000	558,000	1,000,000	4,490,000
		36.4 *	226	532	837	6350	10,300	47,800	107,000	532,000	970,000	4,770,000
	Mean	107	219	505	961	5613	10,865	50,175	102,550	523,750	990,000	4,675,000
	SD	10.9	13.9	83.4	122	492	1340	7110	7722	26,738	17,321	137,961
	%CV	10.1	6.3	16.5	12.6	8.8	12.3	14.2	7.5	5.1	1.7	3.0
	%RE	7.3	−12.4	0.9	−3.9	12.3	8.7	0.4	2.6	4.8	−1.0	−6.5

* Out of acceptance criteria and not included in statistics.

**Table 3:** Summary statistics of intra- and inter-batch precision and accuracy of vitreous humor immunocapture-LC-MS/MS assays.

Monkey Vitreous QCs												
	Low-Concentration Assay						High-Concentration Assay					
	QC low		QC medium		QC high		QC low		QC medium		QC high	
	7.49 ng/mL		30.0 ng/mL		300 ng/mL		300 ng/mL		5000 ng/mL		30,000 ng/mL	
batch	1	2	1	2	1	2	1	2	1	2	1	2
Rep1	6.19	8.22	33.0	23.0	370	296	299	361	6040	5390	32,800	34,800
Rep2	6.23	6.41	32.9	27.6	340	282	338	356	5130	5620	35,000	34,100
Rep3	6.06	ND	31.7	26.5	342	284	327	328	5480	5550	34,300	33,300
Rep4	5.61	7.91	30.6	26.7	343	311	354	322	5310	5800	35,600	35,100
Intra-Batch Summary Statistics												
mean	6.02	7.51	32.1	25.9	349	293	330	342	5490	5590	34,425	34,325
SD ^a^	0.29	0.97	1.14	2.01	14.3	13.1	23.2	19.6	394	170	1207	802
%CV ^b^	4.8%	12.9%	3.5%	7.7%	4.1%	4.5%	7.0%	5.7%	7.2%	3.0%	3.5%	2.3%
%RE ^c^	−19.6%	0.3%	6.9%	−13.5%	16.2%	−2.3%	9.8%	13.9%	9.8%	11.8%	14.8%	14.4%
n	4	4	4	4	4	4	4	4	4	4	4	4
Inter-Batch Summary Statistics												
mean	6.66		29.0		321		336		5540		34,375	
SD	0.99		3.60		32.3		20.9		286		950	
%CV	14.9%		12.4%		10.1%		6.2%		5.2%		2.8%	
%RE	−11.1%		−3.3%		7.0%		11.9%		10.8%		14.6%	
n	8		8		8		8		8		8	

^a^ Standard deviation. ^b^ Coefficient of variation. ^c^ Relative error.

**Table 4:** Summary statistics of intra- and inter-batch precision and accuracy of aqueous humor immunocapture-LC-MS/MS assays.

**Aqueous Humor QCs**												
	**Rabbit Aqueous**						**Monkey Aqueous**					
	QC Low		QC Medium		QC High		QC Low		QC Medium		QC High	
	300 ng/mL		5000 ng/mL		30,000 ng/mL		300 ng/mL		5000 ng/mL		30,000 ng/mL	
Batch	1	2	1	2	1	2	1	2	1	2	1	2
Rep1	378 *	365	5930	5780	33,800	33,000	376	371	6250	6210	37,100	35,800
Rep2	371	373	5820	5830	32,000	33,300	352	363	6000	6010	34,500	36,600
Rep3	318	382 *	5790	5830	33,900	32,100	322	381 *	5330	6020	33,000	31,800
Rep4	361	352	5580	5580	33,200	31,900	356	359	5490	5820	34,100	32,400
Intra-Batch Summary Statistics												
Mean	350	363	5780	5755	33,225	32,575	352	364	5768	6015	34,675	34,150
SD ^a^	28.2	10.6	146	119	873	680	22.3	6.1	430	159	1737	2402
%CV ^b^	8.0%	2.9%	2.5%	2.1%	2.6%	2.1%	6.3%	1.7%	7.5%	2.6%	5.0%	7.0%
%RE ^c^	16.7%	21.1%	15.6%	15.1%	10.8%	8.6%	17.2%	21.4%	15.4%	20.3%	15.6%	13.8%
n	2	2	2	2	2	2	4	4	4	4	4	4
Inter-Batch Summary Statistics												
Mean	357		5768		32,900		357		5891		34,413	
SD	20.4		124		804		17.5		328		1961	
%CV	5.7%		2.2%		2.4%		4.9%		5.6%		5.7%	
%RE	18.9%		15.4%		9.7%		19.0%		17.8%		14.7%	
n	4		4		4		4		4		4	

* Out of acceptance criteria. ^a^ Standard deviation. ^b^ Coefficient of variation. ^c^ Relative error.

**Table 5:** Summary statistics of intra- and inter-batch precision and accuracy of retina tissue immunocapture-LC-MS/MS assays.

Monkey Retina Tissue QCs						
	QC Low		QC Medium		QC High	
	300 ng/g-Tissue		25,000 ng/g-Tissue		3,750,000 ng/g-Tissue	
batch	1	2	1	2	1	2
Rep1	471 *	187 *	23,400	28,400	3,770,000	4,080,000
Rep2	257	189 *	27,800	27,300	3,160,000	3,860,000
Rep3	359	223	25,200	28,000	3,320,000	4,730,000
Rep4	328	213	27,700	26,300	3,950,000	3,620,000
Intra-Batch Summary Statistics						
mean	315	218	26,025	27,500	3,550,000	4,072,500
SD *^a^*	52.3	7.07	2123	920	371,214	476,891
%CV *^b^*	16.6%	3.2%	8.2%	3.3%	10.5%	11.7%
%RE *^c^*	4.9%	−27.3%	4.1%	10.0%	−5.3%	8.6%
n	3	2	4	4	4	4
Inter-Batch Summary Statistics						
mean	276.00		26,762.5		3,811,250	
SD	64.7		1708		484,280	
%CV	23.4%		6.4%		12.7%	
%RE	−8.0%		7.1%		1.6%	
n	5		8		8	

* Out of acceptance criteria. *^a^* Standard deviation. *^b^* Coefficient of variation. *^c^* Relative error.

**Table 6:** List of all bioanalytical assays in support of the monkey ocular PK study.

	Immunocapture-LC/MS/MS					ELISA
Assay Parameter	Plasma	Vitreous Humor		Aqueous Humor	Retina	Aqueous Humor Albumin ELISA
		Low Concentration	High Concentration			
Immunocapture	Protein/peptide	Protein	Protein	Peptide	Peptide	n/a
Sample volume	500 µL	250 µL	10 µL	25 µL	50 µL homogenate	5 µL
Digestion time	Overnight	2 h	2 h	2 h	2 h	n/a
Linear Range	0.05–100 ng/mL	1.26—500 ng/mL	100–50,000 µg/mL	10–50,000 ng/mL	0.1–5000 µg/g tissue	6.25 to 800 ng/mL

## Data Availability

Data are contained within the article.
